# On the rules of continuity and symmetry for the data quality of street networks

**DOI:** 10.1371/journal.pone.0200334

**Published:** 2018-07-12

**Authors:** Xiang Zhang, Weijun Yin, Shouqian Huang, Jianwei Yu, Zhongheng Wu, Tinghua Ai

**Affiliations:** 1 School of Resource and Environmental Science, Wuhan University, Wuhan, China; 2 State Key Laboratory of Information Engineering in Surveying Mapping and Remote Sensing, Wuhan University, Wuhan, China; 3 Hi-Target Surveying Instrument Co., Ltd., Guangzhou, China; 4 Wuhan Hi-Target Digital Cloud Technology Co., Ltd., Wuhan, China; 5 NavInfo Co., Ltd., Wuhan, China; University of South Carolina, UNITED STATES

## Abstract

Knowledge or rule-based approaches are needed for quality assessment and assurance in professional or crowdsourced geographic data. Nevertheless, many types of geographic knowledge are statistical in nature and are therefore difficult to derive rules that are meaningful for this purpose. The rules of continuity and symmetry considered in this paper can be thought of as two concrete forms of the first law of geography, which may be used to formulate quality measures at the individual level without referring to ground truth. It is not clear, however, how much the rules can be faithful. Hence, the main objective is to test if the rules are consistent with street network data over the world. Specifically, for the rule of continuity we identify natural streets that connect smoothly in a network, and measure the spatial order of information (e.g. names, highway level, speed, etc.) along the streets. The measure is based on spatial auto-correlation indicators adapted for one dimension. For the rule of symmetry, we device an algorithm that recognize parallel road pairs (e.g. dual carriageways), and examine to what extent attributes in the pairs are identical. The two rules are tested against 28 cities selected from OpenStreetMap data worldwide; two professional data sets are used to show more insights. We found that the rules are consistent with street networks from a wide range of cities of different characteristics, and also noted cases with varying degrees of agreement. As a side-effect, we discussed possible limitations of the autocorrelation indicators used, where cautions are needed when interpreting the results. In addition, we present techniques that performed the tests automatically, which can be applied to new data to further verify (or falsify) our findings, or extended as quality assurance tools to detect data items that do not satisfy the rules and to suggest possible corrections according to the rules.

## Introduction

Crowdsourced geographic information or volunteered geographic information (VGI) [1] is an important source for gathering data/facts about our world, complementary to the traditional data providers like national mapping agencies and related companies. Currently it has become the basis of numerous applications in the public domain such as information services, knowledge discovery, and indoor/outdoor navigation [2–4], and is playing an increasingly bigger role in providing a creative and quantitative research framework for social and environmental sciences [5–8]. OpenStreetMap (OSM) is one of the most prominent crowdsourcing projects that collects geographic data by citizens. However, crowdsourced geographic information is constantly suffering from quality issues. This includes the semi-controlled vocabulary used in the tagging system, which makes it easy to lend itself to creative usages (see e.g. http://wheelmap.org) but also makes the data more vulnerable. OSM for example adopts a lazy evaluation approach, where erroneous data may be registered into the database before users identify and correct the errors. This is one of the keys to OSM’s success [9], but the correction can take long depending on how popular the data is in use, and some errors may never be found if they locate in remote, less populated areas [10].

In general, quality assessments in VGI can be divided into two categories: ones that are independent and rely on rules and knowledge (intrinsic) and ones that have to refer to external ground-truth data (extrinsic). Extensive studies have focused on the use of extrinsic assessments to evaluate the overall quality of VGI datasets [11, 12]. In the process ground-truth data must be present, which could be both expensive and often unavailable, either for certain regions or for large scale quality evaluations. If the evaluations are to be carried out at a finer level (e.g. for individual features), features in testing and reference datasets should be matched in the first place [13, 14]. Feature matching is itself a challenging problem [15, 16]. Hence intrinsic quality assessments are sought for to overcome the limitations of extrinsic approaches. Trust was used as an indicator to estimate the data quality of VGI without comparisons to third party data with proven quality [17, 18]. This is based on the assumption of Linus’s Law [19] and, in OSM, indicators such as the number of versions, users, corrections available in the editing history was used to indirectly reflect OSM quality. However, although trust may give a general impression of how quality is distributed across the data, it may fall short in predicting how the data is problematic (whether it is in completeness, accuracy, consistency, etc.), and manual scrutiny is necessary to further identify the erroneous items. On the other hand, Goodchild & Li [10] noted that such a social approach may fail for facts that are not prominent, or regions that lack contributors of sufficient local knowledge. Empirical studies also reveal that, though the positional accuracy improved as the number of contributors increases up to 13, the number was not strongly related to the data quality for ‘heavily edited’ objects [19, 20]. In short, the trust-based approach is still open to further verification.

On the contrary, we look for intrinsic quality measures from a physical perspective. In this respect, Goodchild & Li [10] discussed the use of geographic knowledge for verifying the information contributed by citizens. They tested the use of fractal laws of linear features [21], Horton’s law of channels [22] as well as Central Place Theory in economic geography [23]. But they found that the laws failed to distinguish imaginary landscapes (in drawing arts) from our real world, which we believe is that the laws are so much general so that they may also apply for landscapes out of our planet, and that the imaginary land happens to capture their essential properties. The first two laws are actually scaling laws that can be observed more broadly in fields outside geography.

More recently, complex network approaches in structured sociology and statistical mechanics have been introduced to study spatial networks in urban systems [24]. This leads to the findings of small-world and/or scale-free networks in geography such as street networks. However, such scaling laws are statistical in nature and are not suitable for developing quality assurance tools on top of them. For example, even if we know that the length or connectivity of natural streets is power-law distributed [24, 25], there is no way to know the accuracy or consistency of a single street. This is because the power law distribution emerges only when the number of streets becomes huge and a number of exceptions do not change the appeared distribution.

In this study, we aim to inspect quality assurance rules that can be observed and used at the individual level rather than statistical rules for the entire data. The rules are concrete forms of Tobler’s First Law (TFL) of geography [26]. In addition to giving clues to where the data is problematic, the rules should be able to notify users of possible corrections.

### The geometry of road networks

**Fig 1:**
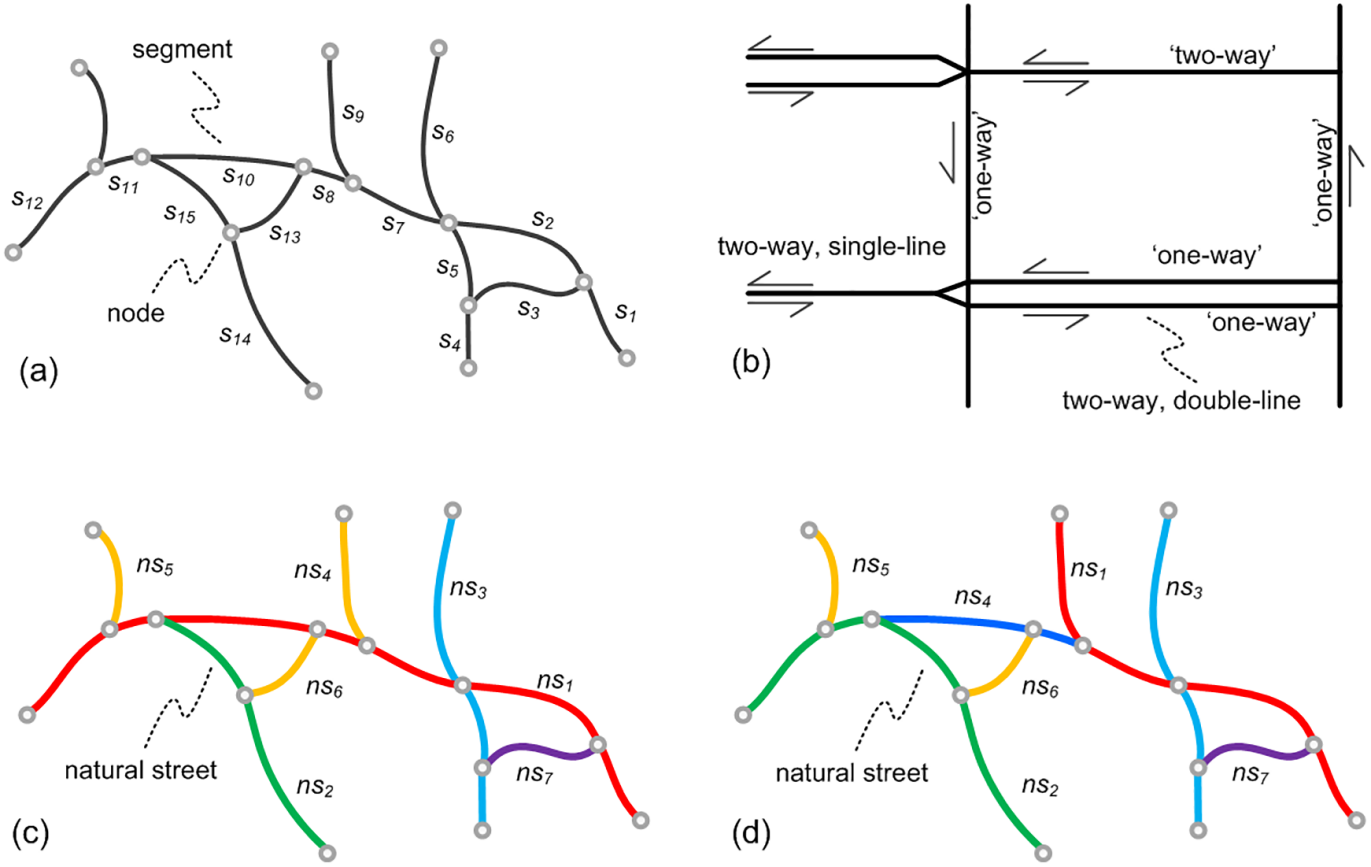
**Geometry of road networks and related concepts demonstrated**: nodes and segments (a); one-way, two-way streets, single-line, and double-line representations (b) natural streets (in the same color) formed by every-best-fit (c) and self-best-fit (d) strategies.

To disambiguate the terms used in the paper, we make some clarifications here. A road/street is a passage that can be travelled either in a single direction (i.e. one-way road) or in two directions (i.e. two-way road). Technically, a street consists of consecutive segments which are the atomic units for tagging and annotation in data and are bounded by two nodes (Fig 1(a)). A two-way road (e.g. dual carriageway or divided highway) can either be modeled by a single line or double lines (Fig 1(b)). The symmetry rule will mainly examine the two-way roads modeled by double lines.

A natural street is a path of street segments that are connected based on the principle of good continuity [25]. It reflects the self-organizing nature in street networks. The principle has been in use for more than a decade in cartography and urban geography for delineating the structure of street networks [27, 28], and was later adapted for analyzing the scaling of geographic phenomena using complex network approaches [24, 29, 30]. In short, natural streets can be formed by starting from a segment and connecting smoothly the next segment until the angle of deflection in the connection exceeds a certain threshold. Two methods to recognizing natural streets (Fig 1(c) and 1(d)) will be outlined in Forming of natural streets.

Likewise, named streets are consecutive segments with the same name [31]. The concept corresponds directly to geographic entities of roads but are harder to recognize than natural streets due to the missing or incorrect names [25]. This is especially the case in OSM data. Note that, named streets can be related to natural streets because commonly the former is contained in the later [32]. For example, if we assume that segments {*s*_1_, *s*_2_, *s*_7_, *s*_8_} are named ‘A’ and {*s*_10_, *s*_11_, *s*_12_} named ‘B’, then both named streets are contained in the natural street *ns*_1_ (Fig 1(c)). This constitutes the basis of using natural streets as a rule-of-thumb to inspect the quality of street networks like missing and incorrect names [32].

### Hypothesis

**Fig 2:**
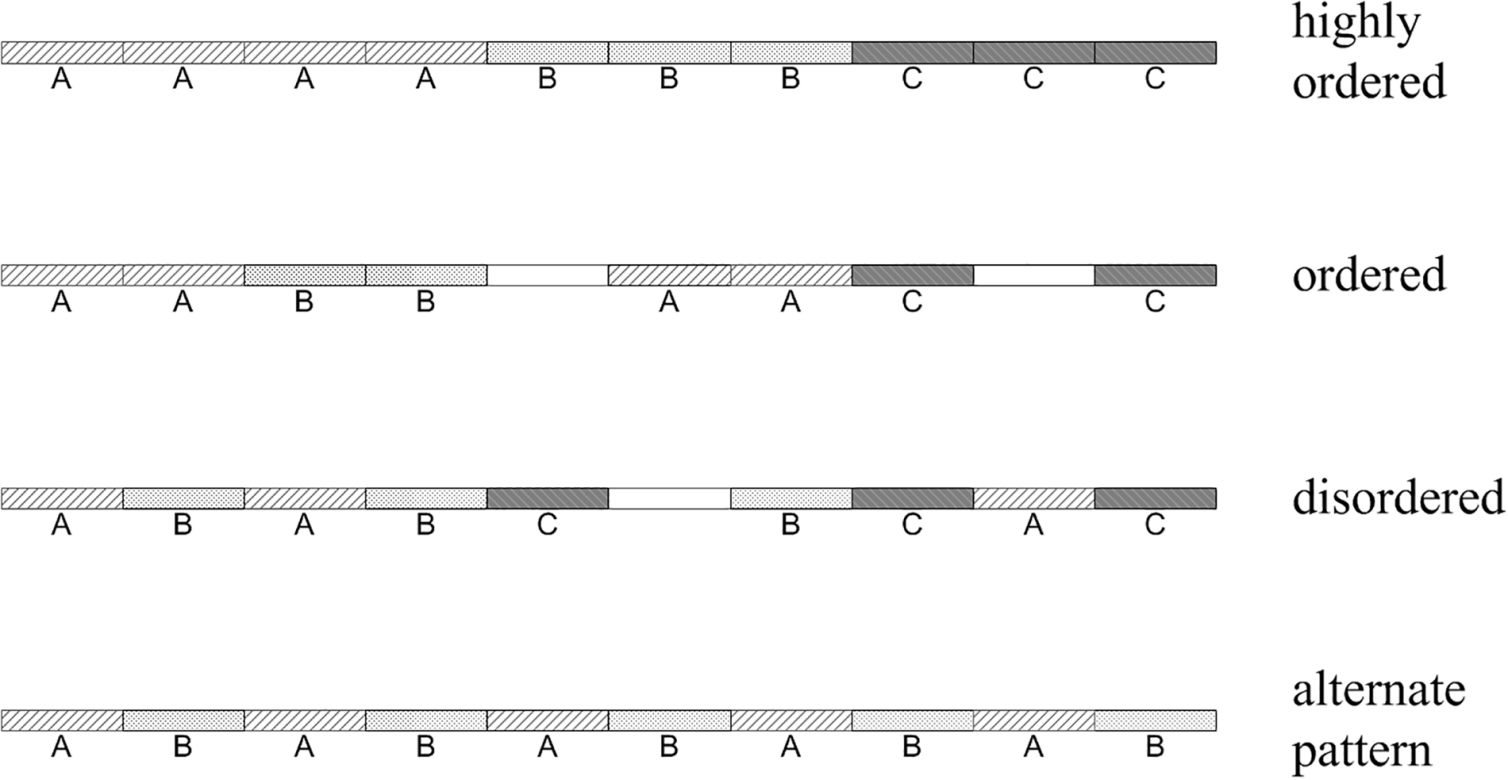
Diagram illustrating degrees of spatial order of information along a continuous group of street segments. A, B and C are three values of an attribute attached to the segments; white blocks indicate segments with empty values.

The two rules considered here are concrete forms of TFL of geography. A starting point of our hypothesis is that, the names of segments in a named street should be the same. With this principle, we are able to identify the missing or incorrect names. However, as we are currently only able to detect natural streets, the principle should somehow be relaxed. It is hypothesized as the rule of continuity, and is extended to include information types other than street names. This rule can be formulated as: information (attributes assigned to segments) is continuously distributed along natural streets, presenting a high level of spatial order (Fig 2(a)). One of our aims is to find out how this rule is consistent with street networks over the world.

As for the rule of symmetry, we refer to the hypothesis that attribute values on the opposite sides of a double-line street mirror each other (a property of symmetry). For example, the street name, highway class, speed limit, etc. should be probably the same or similar and traffic directions in reverse. In this paper we aim to find out how widely this rule of symmetry can be observed and for which types of information.

## Materials and methods

### Measures of spatial order along natural streets

#### Forming of natural streets

Basically, a natural street is formed by starting from a segment and connecting smoothly the next segment, this process continues until no segment can be smoothly connected [27]. The smoothness in the connection can be defined in different ways, and we present two main strategies: self-best-fit and every-best-fit [25]. In the self-best-fit strategy, the algorithm finds the connecting segment with the smallest deflection angle (*θ*) during the search, and if this angle is smaller than a certain threshold (e.g. *θ* ≤ 60°), this segment is the self-best-fit of current segment. In the every-best-fit strategy, current segment first searches for a self-best-fitted segment, and this segment is connected only if current segment is also a self-best-fit of it. So at junctions with a degree larger than 2, the every-best-fit strategy always looks for the optimal smooth connection (Fig 1(c)), while the result from self-best-fit depends on the sequence of input segments (Fig 1(d)). For a detailed discussion of the strategies, readers are referred to [25].

The appeared spatial order along natural streets will be influenced by the strategy chosen. For instance, the above-mentioned two named streets {*s*_1_, *s*_2_, *s*_7_, *s*_8_} and {*s*_10_, *s*_11_, *s*_12_} are contained in the same natural street formed by every-best-fit, but are segmented into pieces by the self-best-fit strategy (c.f. Fig 1(c) and 1(d)). We use the every-best-fit strategy because it leads to a unique configuration of natural streets, which maximize the spatial order that can be observed in the street networks (for a more quantitative analysis see Alternative strategies for continuous segments).

#### Degrees of spatial order

Fig 2 illustrates degrees of spatial order of attribute values along a schematic natural street. Measures should be able to characterize these different degrees. Several measures may be related. Information theoretic approaches to spatial data have been developed for measuring the information content (entropy) of maps of many sorts [33, 34]. To measure the degree of order in spatial data, spatial autocorrelation is considered in Bjørke [34] to reformulate the entropy computation. Nevertheless, it seems that the adapted measure is not able to characterize alternate patterns in Fig 2(d) as the equation becomes undefined then.

To measure the spatial order along natural streets and to characterize different patterns in Fig 2, we use measures of spatial autocorrelation, i.e., join-count statistic (JCS) for qualitative data and Moran’s *I* [35] for quantitative data.

#### Join-count statistic (JCS) for qualitative data

Join-count statistic is a way of measuring the degree of clustering or dispersion when the variable is qualitative (street name, highway class, etc.). In our work, textual values that cannot be rank-ordered fall into this category. JCS counts the number of joins (connections) of the same value (*J*_*rr*_) and that of different values (*J*_*rs*_) along a natural street, and then compares them with expected joins ($E[J_{rr}]$ and $E[J_{rs}]$) under the random assumption. Note that *J*_*rr*_ and *J*_*rs*_ are computed for every *r* and *s* value in a natural street. Take the highly ordered street in Fig 2 for example, we would have *J*_*rr*_ = {3, 2, 2} for A, B, C values respectively, and *J*_*rs*_ = 2 for this street (i.e. the joins of AB and BC). If $J_{rr}>E\left[J_{rr}\right]$ and $J_{rs}<E\left[J_{rs}\right]$, the data appears to be positively spatial autocorrelated (i.e. similar values appear clustered in space). Cliff & Ord [36] proved that the joins follows an asymptotically Gaussian distribution and gave a numerical derivation of the mean and variance. These are commonly used for testing the significance of the results. In the following, we present the JCS formulation and derive some reduced forms that are suitable for one dimensional cases, and we assume sampling without replacement as it is more realistic for geographic properties.

First of all, several quantities related to JCS are defined on the connectivity (or weight) matrix, which has a special form for one dimensional cases like natural streets (e.g. Table 1). The expected number of joins of the same value is given by Cliff & Ord [36]:

$$E\left[J_{rr}\right]=\frac{Wn_{r}\left(n_{r}-1\right)}{2N(N-1)}\;,$$ (1)

where *N* is the number of road segments in a natural street, *n*_*r*_ is the number of segments that are of the same value *r*, and

$$W=\sum_{i}\sum_{j}w_{ij}\;,$$ (2)

which can be reduced in our one dimensional case to:

$$W=\{\begin{array}{cc} 2(N-1), & \text{if}\;\text{the}\;\text{street}\;\text{is}\;\text{open}\\ 2N, & \text{if}\;\text{it}\;\text{is}\;\text{closed}\;\text{(first}\;\text{and}\;\text{last}\;\text{nodes}\;\text{coincide)} \end{array}$$ (3)

**Table 1:** The connectivity matrix for a natural street of 8 segments.

	1	2	3	4	5	6	7	8	∑
1	0	1	0	0	0	0	0	0	1
2	1	0	1	0	0	0	0	0	2
3	0	1	0	1	0	0	0	0	2
4	0	0	1	0	1	0	0	0	2
5	0	0	0	1	0	1	0	0	2
6	0	0	0	0	1	0	1	0	2
7	0	0	0	0	0	1	0	1	2
8	0	0	0	0	0	0	1	0	1
∑	1	2	2	2	2	2	2	1	14

The variance of observing *J*_*rr*_ under the null hypothesis of spatial randomness is:

$$\begin{array}{cc} \sigma {\left[J_{rr}\right]}^{2}= & \frac{S_{1}n_{r}\left(n_{r}-1\right)}{4N(N-1)}+\frac{\left(S_{2}-2S_{1}\right)n_{r}\left(n_{r}-1\right)\left(n_{r}-2\right)}{4N(N-1)(N-2)}\\ & +\frac{\left(W^{2}+S_{1}-S_{2}\right)n_{r}\left(n_{r}-1\right)\left(n_{r}-2\right)\left(n_{r}-3\right)}{4N(N-1)(N-2)(N-3)}\\ & -E{\left[J_{rr}\right]}^{2}\;, \end{array}$$ (4)

where

$$S_{1}=\frac{1}{2}\sum_{i}\sum_{j}{\left(w_{ij}+w_{ji}\right)}^{2}$$ (5)

Because the connectivity matrix is symmetric in our case, it implies *w*_*ij*_ = *w*_*ji*_. The above equation can be reduced to $S_{1}=2\sum_{i}\sum_{j}w_{ij}^{2}$, and since *w*_*ij*_ equals to either 1 or 0, *S*_1_ = 2∑_*i*_∑_*j*_ *w*_*ij*_ = 2*W*.

$$S_{2}=\sum_{i}(\sum_{j}w_{ij}+\sum_{j}w_{ji}{)}^{2}$$ (6)

By observing the connectivity matrix (Table 1) for one dimensional open sequences, *S*_2_ can be rewritten here as:

$$\begin{array}{cc} S_{2} & ={\left(1+1\right)}^{2}+\underset{N-2}{\underset{︸}{{\left(2+2\right)}^{2}+{\left(2+2\right)}^{2}+\cdots +{\left(2+2\right)}^{2}}}+{\left(1+1\right)}^{2}\\ & =2{\left(1+1\right)}^{2}+\left(N-2\right){\left(2+2\right)}^{2}\\ & =16N-24\;, \end{array}$$ (7)

where it requires that the number of units in any open sequence satisfies *N* ≥ 2. Likewise, we can obtain *S*_2_ = 16*N* for any closed sequence (*N* > 2).

For expected number of joins of different values ($E[J_{rs}]$) and its variance under random assumption (*σ*[*J*_*rs*_]^2^), we use the original formula under the assumption of sampling without replacement [36], and replace the terms *W*, *S*_1_, *S*_2_ with their reduced form as presented above. Note that, in order for *σ*[*J*_*rs*_]^2^ to be meaningful, it requires that a natural street consists of at least 4 segments (*N* ≥ 4).

Z-test is used here for significance testing: $z_{rr}=\left(J_{rr}-E\left[J_{rr}\right]\right)/\sqrt{\sigma {\left[J_{rr}\right]}^{2}}$. The testing for joins of different values (*z*_*rs*_) is formulated in a similar way. Note that if all segments in a natural street are of the same value (i.e. *N* = *n*_*r*_), *z*-score is undefined because Eq (4) equals to zero. This should be interpreted as the strongest form of spatial order.

#### Moran’s *I* for quantitative data

Moran’s *I* is a measure of spatial autocorrelation for numerical values. In our work attribute values with a defined mean such as speed limits fall into this category. We use standard derivations to calculate the *I* index and its variance [35]. As in our case, Moran’s *I* shares the same connectivity matrix with JCS, so we replace the terms *W*, *S*_1_, *S*_2_ in the standard formula with their reduced form as presented above. The calculated *I* ranges from -1 that indicates negative autocorrelation to 1 that indicates positive autocorrelation. Z-test is used for significance testing as well.

### Measures of symmetrical order in parallel streets

First we consider divided highways (or dual carriageways) to be typical examples of this rule. But technically we are not able to precisely identify such highways as such divided highways are usually not encoded in the data. So we rely purely on the geometry to recognize parallel streets that are not too wide in a restricted network and, in this way, we make sure that divided highways are included.

#### Measures of parallelism in streets

Due to the difficulty in recognizing divided highways, we focus on parallel streets in the main roads in a city (e.g. tertiary, secondary, primary, trunk roads and motorways in OSM), where divided highways most likely occur. By restricting the network to a smaller, more backbone network, the ambiguities in detecting divided highways can be reduced.

**Fig 3:**
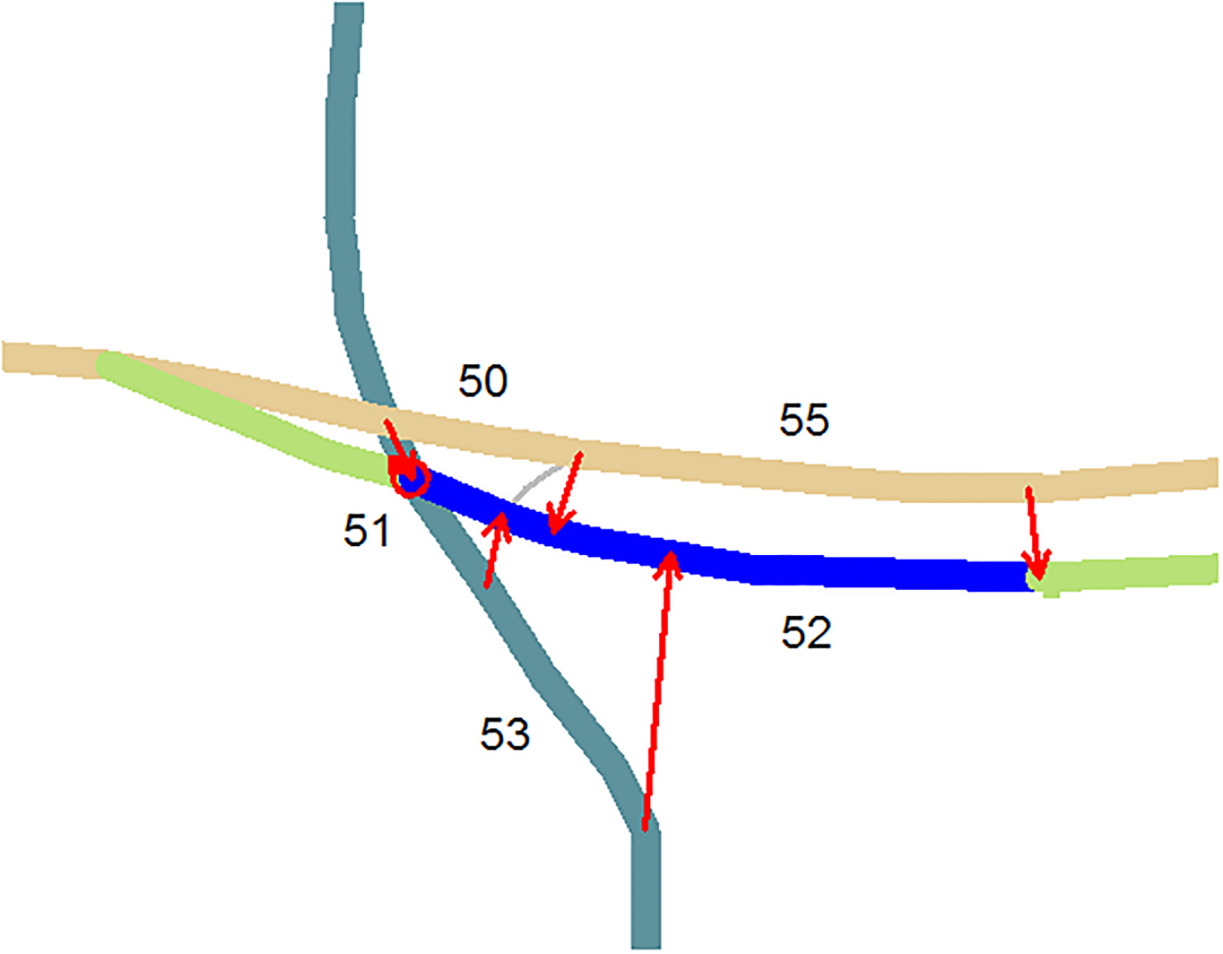
Measures of parallel streets in an intersection. The blue line is the source road; arrows indicate the part of the two roads for which the parallelism are measured; roads are numbered in the same order as in Table 2.

Ideally, parallel curves can be defined as being everywhere equally distant from each other. This means that the nearest distance between points on two curves are everywhere the same. In real data however, road segments on the opposite side of a divided highway are not always perfectly parallel to each other. Even for perfect parallel roads, the methods used for data acquisition and sampling also introduce noises that may further obscure our distance-based analysis. Hence the inter-distance for parallel curves is not constant, but may fluctuate around some mean value. A reasonable assumption is that the distance is uniform with normally distributed noises. As a result, the mean (*μ*_*dis*_) and standard deviation (*σ*_*dis*_) of distances and their coefficient of variance (*cv*_*dis*_ = *σ*_*dis*_/*μ*_*dis*_) can be used to characterize the degree of parallelism between two discrete curves. Fig 3 demonstrates several road segments in an intersection, with the measured parallelism shown in Table 2.

**Table 2:** Measured parallelism corresponding to the road pairs in Fig 3.

source id	candidate id	*σ*_*dis*_	*μ*_*dis*_	*cv*_*dis*_
52	50	1.757	11.325	0.155
	51	4.824	4.709	1.024
	53	10.816	24.173	0.447
	55	0.669	14.840	0.045

Note that mean distance *μ*_*dis*_ should be useful in distinguishing parallel roads that are part of a dual carriageway from those that are not. However, there seems to be no widely acceptable value that can be used for all cities (see The algorithm & parameters). We used thresholds $T_{\mu_{dis}}=60m$, *T*_*cv*_ = 0.2 for this study: any pair of road segments that does not exceed $T_{\mu_{dis}}$ and *T*_*cv*_ is considered a divided highway (or parallel street).

#### A procedure to detect parallel roads

Practically, parallelism is measured between individual segments rather than natural streets. To ensure the robustness of the parallelism statistics, we insert more points to the original segments at an interval equal to the minimum distance between the segments in the data. This has also an advantage that longer parallel parts on the streets have more weight and hence the parallel testing is more stable under small variations.

**Fig 4:**
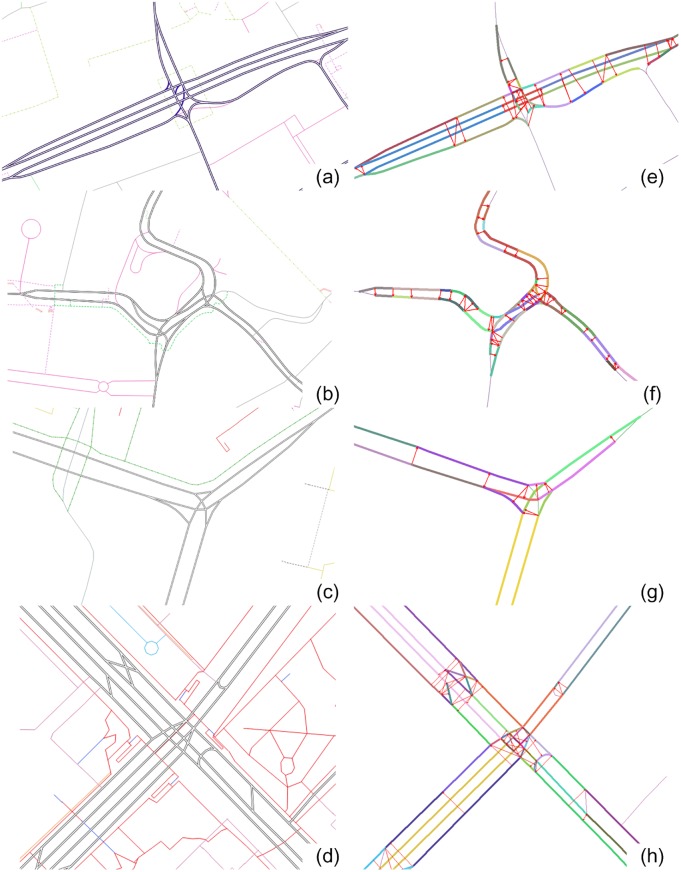
Parallel streets detected in road intersections. Situations before (left) and after the parallel road detection (right): recognized parallel streets usually have the same color code though not always so for those that pairs with more than one segments; arrows further indicates for which part two streets are in parallel.

The distance-based parallel measures can be extremely time-consuming, and therefore we adopt here simple filters that quickly reduce the number of candidates that are then used for the subsequent parallelism testing. The procedure includes the following steps: (1) candidate searching; (2) anchor points adjustment: this is to identify which parts of the road parallel to each other (see red links in Fig 3 for anchor points); (3) end points testing: to remove pairs that are apparently not parallel; (4) parallelism testing: all points (including the inserted ones) between the anchor points are used to measure the parallelism. For the first three steps, one can refer to [37] for more details. Parallel streets detected in real highway intersections are exemplified in Fig 4.

## Experimental settings

We tested the rules against 28 cities in OSM data and 2 professional data sets (Table 3). These cities are carefully selected so that they cover different characteristics: patterns of street networks, metropolitan/urban/rural areas, left/right-riding countries, countries of different cultures and languages. For OSM data we focus on the most commonly used 7 attributes: ‘name’, ‘highway’, ‘ref’ (reference number), ‘maxspeed’, ‘oneway’, ‘bridge’, and ‘bicycle’, where the latter ones are less commonly used than the first two. Professional data sets include an Ordnance Survey Open Map data (London-OS) and a navigation data (Nav), which do not have attributes identical to those of OSM. So we tested some attributes as close to the OSM ones as possible.

**Table 3:** Summary of natural streets (i.e. strokes) in each data set and the significance of spatial autocorrelation (*z*-scores) of the ‘name’ attribute in natural streets by join-count statistic.

		(%)			(%)	for strokes having *N* ≥ 10 segments			
city	#roads	$\frac{\text{name}=\text{null}}{\#\text{road}}$	# strokes	# strokes used	undefined *z*_*rr*_	*μ*(*z*_*rr*_)	*σ*(*z*_*rr*_)	*μ*(*z*_*rs*_)	*σ*(*z*_*rs*_)
London-OS	327885	6.30	104035	21944	50.40	4.09	1.94	-7.52	4.24
Nav	66697	0.00	8153	3079	80.32	6.83	4.30	-13.29	7.86
Ahmedabad	7761	86.30	5513	202	50.00	0.04	1.98	NULL	NULL
Amsterdam	63665	47.50	23133	3994	38.00	2.85	1.56	-4.65	2.62
Athens	61555	47.70	38254	2368	28.90	3.05	1.55	-4.90	2.62
Bangkok	93495	89.40	76927	1193	58.60	2.94	1.85	-5.11	2.92
Barcelona	18457	39.70	9603	1001	35.00	3.00	1.03	-4.63	1.54
Beijing	52327	77.10	31296	1767	41.54	2.26	2.77	-3.17	3.77
Cario	40771	79.90	31279	639	56.70	2.13	1.43	-3.95	1.93
Frankfurt	21233	63.50	4867	523	33.30	3.72	1.79	-6.63	4.26
Geldermalsen	35438	35.40	15853	2264	31.80	2.91	1.11	-4.82	2.15
Hong Kong	53251	67.70	31291	2330	50.30	3.16	1.21	-5.52	2.30
Istanbul	27174	86.00	21233	539	55.50	2.06	1.70	-4.43	1.99
London-core	27983	48.00	15503	1288	34.60	3.34	1.54	-5.81	3.24
Moscow	199382	89.70	128267	6618	77.10	4.43	3.07	-8.00	9.46
Nagasaki	33846	92.00	11605	2629	93.40	3.41	2.12	-6.14	3.87
Nicosia	9604	55.30	7249	168	37.50	1.96	1.16	-3.95	0.74
Norwich	26169	55.40	15115	968	56.80	3.94	1.91	-7.00	3.94
Ottawa	45879	42.20	25265	1998	41.80	3.51	1.65	-5.62	2.70
Paris	28906	48.30	15481	1487	38.00	2.97	1.30	-4.77	2.63
Rio	24269	45.40	16507	729	22.40	3.14	1.32	-5.14	2.42
Santiago	67148	23.90	52529	1265	27.20	3.08	1.46	-4.99	2.31
Seattle	119437	51.50	79375	3198	36.50	4.46	2.51	-6.96	4.50
San Francisco	246327	40.50	155289	6320	37.50	4.60	2.81	-7.12	5.41
Shanghai	53441	58.50	25488	2530	62.50	3.70	2.25	-6.11	3.74
Singapore	42827	54.20	28546	1203	32.00	3.35	1.73	-5.44	3.69
Sydney	33004	36.40	19410	1392	39.00	3.45	1.48	-5.51	3.10
Toronto	157220	50.80	88369	6517	46.90	3.35	1.51	-5.21	2.31
Wellington	8184	64.80	5940	225	22.20	2.57	0.88	-4.42	1.34
Wuhan	17614	75.90	10792	570	48.42	3.05	1.81	-5.08	2.29

To verify the rule of continuity, we distinguished between categorical (e.g. name, highway class) and numerical attribute values (e.g. speed limit). JCS and Moran’s *I* were used where appropriate to test whether attribute values are spatially ordered in the smoothly connected natural streets. If the values show positive spatial auto-correlation with statistical significance (Z-test), it gives strong support to the rule of continuity.

To verify the rule of symmetry, we evaluated the proportion of parallel pairs of segments sharing the same attribute values in relation to all parallel pairs: the higher the proportion the stronger the supporting evidence. The evaluation was carried out for the same set of attributes used for verifying the rule of continuity. To avoid the sampling bias we tested the rule against all pairs of parallel segments which may contain false positives (see Measures of parallelism in streets).

The two procedures are computationally quite intensive. For each city there are about 10*k* ∼ 320*k* road segments. For the rule of continuity, 5*k* ∼ 150*k* natural streets were detected for each of the cities, and JCS and Moran’s *I* statistics were calculated for each natural street (which are on their own demanding). For the rule of symmetry, the parallelism testing was performed between pairs of roads that fall in the vicinity of each other, which is even more demanding than the former procedure. Although the procedures are fully automated, in total we spent over two months to perform the testing (including calibration) on our data sets.

## Results and discussion

### Rule of continuity

#### Alternative strategies for continuous segments

To show that information is more organized (higher level of spatial order) in certain ways, we compare four ways of collecting groups of segments. First, segments are collected randomly from the network (non-continuous random). Second, we ensure that segments are connected linearly, but at each junction we pick up a segment at random (continuous random). Self-best-fit and every-best-fit are two other ways to be tested. First, we counted the number of consecutive segments of the same name in groups of segments collected by the four strategies (Table 4). In every-best-fit, we observe that the length of continuous units of the same name was on average the longest, while in the non-continuous case the average length was one, meaning that no segments of the same name stay next to each other.

**Table 4:** Number of consecutive segments of the same name averaged for four strategies of collecting groups of segments (street data: Nav).

	Non-continuous Random	Continuous Random	Continuous Self-best-fit	Continuous Every-best-fit
# consecutive segments of the same value	1.00	3.03	7.75	7.90

Then, the JCS result (S4A–S4D Fig) suggests further that information organized linearly by the every-best-fit strategy exhibits the highest level of spatial order. The groups formed by the continuous random strategy still appeared mild positive autocorrelation. This is because, when selecting randomly the next segment that connects to current segment, it is likely (at least a 1/3 chance in the case of a 4-way junction) that a smooth connection can be chosen as in the case of the every-best-fit strategy. However, the appeared spatial order in this case is much less significant than in self-best-fit and every-best-fit. When a group of segments is formed by random selection, the tendency of spatial autocorrelation vanished. Taken together, this indicates a unique character that information of street segments is more organized along smoothly connected continuous units, of which the every-best-fit strategy is superior.

#### General results from JCS

First we tested the rule of continuity for qualitative data (e.g. name, highway, ref, etc.) using JCS. The result indicates a high positive spatial autocorrelation across the cities in general, where the mean and variance of *z*_*rr*_ and *z*_*rs*_ for testing the continuity of street names are summarized in Table 3. As it shows, the joins of segments with the same value are significantly more than what could be expected by chance (25 out of 30 data sets are, on average, significant at *p* < 0.01); the joins of segments with different values are significantly less than what could be expected by chance (all data sets are, on average, significant at *p* < 0.001). Moscow, Seattle and San Francisco are among the cities of highest levels of autocorrelation in our OSM data. For professional data which are of high quality, the attribute values along natural streets appear much stronger positive spatial autocorrelation than OSM data. This gives stronger support to the rule of continuity by professional data.

**Fig 5:**
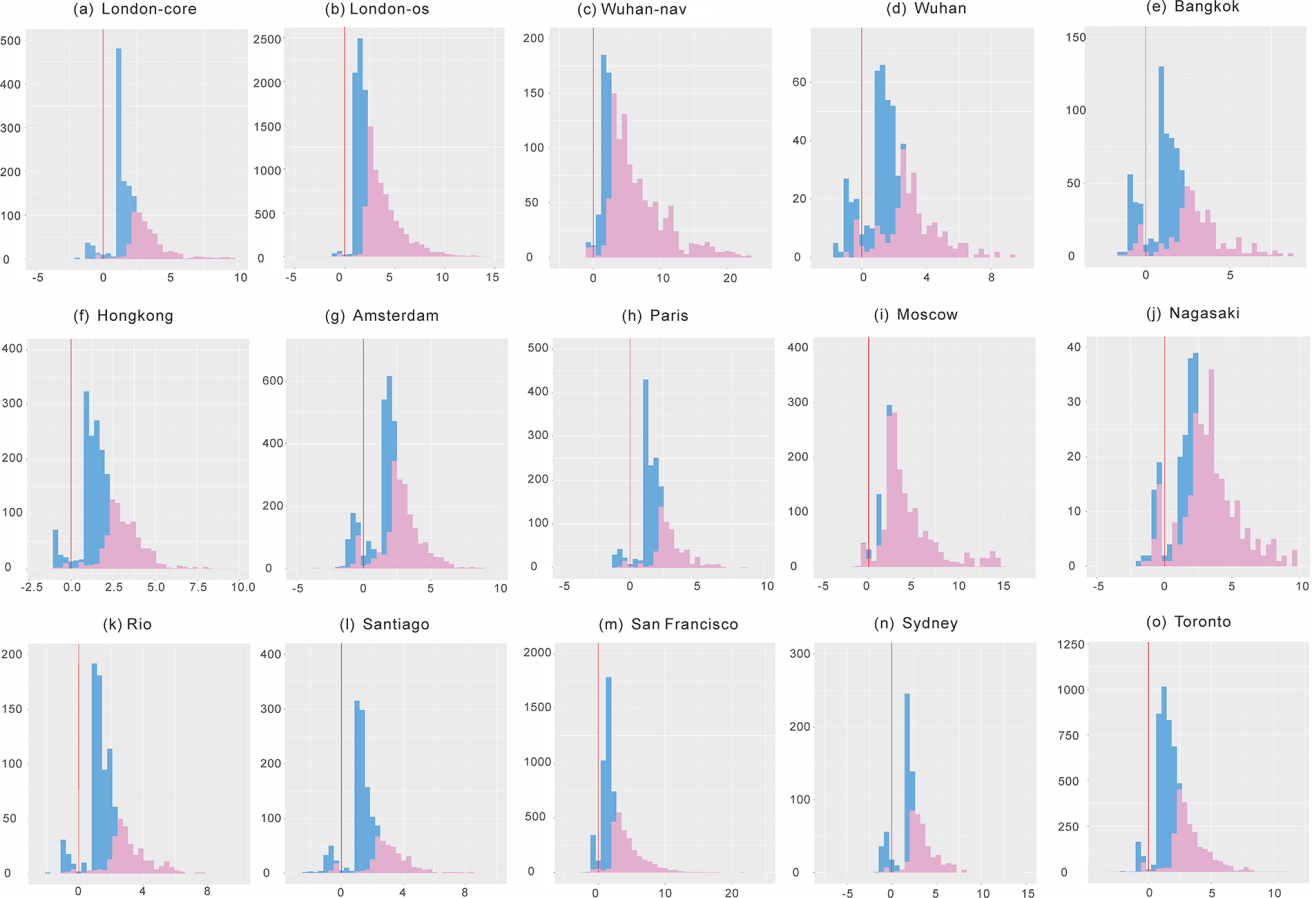
Distributions of *z*_*rr*_ for typical cities. *z*_*rr*_ for all natural streets are shown in blue; *z*_*rr*_ for selected natural streets (*N* ≥ 10) are superimposed on top of the blue ones (red); red vertical line indicates *z* = 0.

As the distributions of *z*_*rr*_ and *z*_*rs*_ within a city are skewed, the mean and variance are not good indicators for a data set. So we show distributions of *z*_*rr*_ for typical cities (Fig 5), which appears that longer natural streets (*N* ≥ 10) have higher *z*-scores.

To get more insights, the distribution of length of natural streets (S5 Fig) is compared. However, despite the fact that both distributions seem to be right-skewed, it is not straightforward to see if the two are correlated. A better view can be seen in S4E–S4H Fig, which indicates that the dependence of JCS on the number of segments (*N*) is conditioned by the way in which the spatial units are organized. Spatial units with large *N* do not necessarily lead to significant JCS (e.g. high *z*_*rr*_). If the spatial units are formed at random (S4A Fig), there is no apparent relation between JCS and *N*; whereas the relation seems to be a bit stronger in S4B Fig. Even with self-best-fit, there are still many cases of large *N* coming with low *z*_*rr*_. Such cases get significantly reduced when natural streets are formed by every-best-fit, suggesting again that the spatial order can be best observed with every-best-fit among the four strategies.

The *z*-scores for joins of segments of different classes (*z*_*rs*_) show a similar pattern (S1 Fig), despite that the values are negative (observed joins less than expected under the random assumption). Because the calculation of *z*_*rs*_ requires natural streets with *N* ≥ 4, the number of the streets involved is much less than those involved in calculating *z*_*rr*_. Large positive *z*_*rr*_ and large negative *z*_*rs*_ values mean that the ‘name’ attribute was positively autocorrelated, which suggests that street names are highly ordered along natural streets.

#### Upper bounds of *z*_*rr*_ values in JCS

Our result indicates that *z*-scores seem to be bounded by *N* if every-best-fit is used. That is, natural streets with fewer segments do not get high *z*-score even if they are highly autocorrelated. Here we try to find a principled explanation for this.

**Fig 6:**
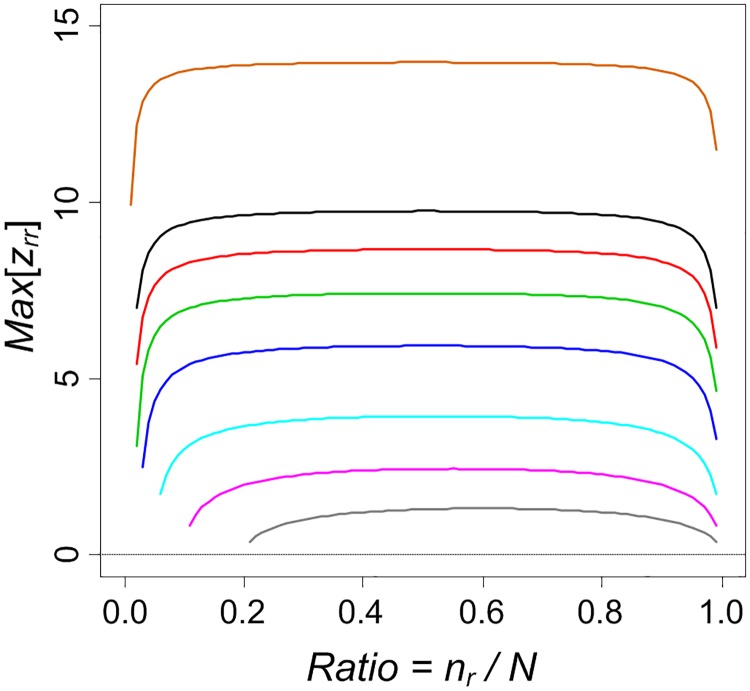
Profiling of theoretical upper bounds of *z*_*rr*_ values. max *z*-scores vary with the ratio *n*_*r*_/*N*; colors indicate *N* = {5, 10, 20, 40, 60, 80, 100, 200} from the bottom to the top.

It can be expected that the strongest form of spatial autocorrelation (i.e. max *z*-score) for one dimensional cases is obtained when the number of joins of the same value *r* satisfies: *J*_*rr*_ = *n*_*r*_ − 1 (first row in Fig 2). In light of Eqs (1) and (4), we speculated that *z*-score for *J*_*rr*_ is somehow related with *n*_*r*_/*N*. So we explore how max *z*-score varies with *n*_*r*_/*N* by simulating a series of *n*_*r*_ and *N*. The simulation is plotted in Fig 6 which shows a high level of regularity.

For one thing, it reveals that the larger the *N* the higher the max *z*-score; the closer the ratio *n*_*r*_/*N* is to 0.5 the higher the max *z*-score. For shorter natural streets (e.g. *N* = 5), the max *z*-score is 1.33 (significance level *p* > 0.05), which can be reached only when *n*_*r*_/*N* ≈ 0.5. As the *z*-score fails to reflect the perceived autocorrelation degree for short natural streets, we focus on natural streets with *N* ≥ 10 in Table 3. This also explains why there are no short natural streets having high *z*-scores (blue bars in Fig 5).

For the other, as *N* increases the range for which max *z*-scores are stable/flat becomes wider. Since this is a multi-class JCS problem, the ratio *n*_*r*_/*N* is usually quite low, especially for longer natural streets. The wide stable range ensures that the resulted *z*-score is not vulnerable to this ratio. Furthermore, the simulation can be used as a look up table: if we know *n*_*r*_ and *N* we know what is the best *z*-score the set of segments in a natural street can achieve (i.e. when segments of the same value stay next to each other, or *J*_*rr*_ = *n*_*r*_ − 1).

#### Distance to the strongest form of spatial order

Although *z*-scores from JCS can indicate the strength of spatial order, a more intuitive way is perhaps to show how close the observed spatial order is to its strongest form (i.e. *Max*[*J*_*rr*_] = *n*_*r*_ − 1). This way, we avoid the small sample limitation in JCS and could study the level of order for all natural streets. First, *z*_*rr*_ is undefined if *n*_*r*_ = *N* which means that the whole natural street is of the same value. This is the strongest form of spatial order (Table 3 suggests that the numbers of natural streets that are in the strongest form of spatial order (undefined *z*_*rr*_) are considerable large for the cities).

For the rest of the natural streets, we compare the observed joins (*J*_*rr*_) with the maximum joins possible (*Max*[*J*_*rr*_]). The result for the typical cities is depicted in S2 Fig. In general, most groups of segments of the same class are in the strongest form of spatial order. That is, observed joins equal to max joins possible (*n*_*r*_ − *J*_*rr*_ = 1). There are also groups of road segments with the same attribute value that are separated into smaller groups (*n*_*r*_ − *J*_*rr*_ > 1). A further inspection reveals that this is mainly due to the presence of empty values which separate the larger group into smaller ones, much like the second row in Fig 2.

#### Negative *z*_*rr*_ values explained

Here we focus specifically on small and negative *z*_*rr*_ values (a small portion of the values left to the vertical lines in Fig 5) to find out if they are exceptions to the rule of continuity. As shown previously, small *z*_*rr*_ values are resulted from short natural streets (*N* ≤ 5) due to the small sample issue of JCS. We then analyzed the negative *z*_*rr*_ for all the cities and found that almost all negative *z*_*rr*_ values can be explained by the following reason: the number of joins of segments with the same attribute value is slightly lower than the max joins (*Max*[*J*_*rr*_] = *n*_*r*_ − 1) by a number of one or two. For this we observed two cases. First, the number of segments with the same value is low (*n*_*r*_ ≤ 5). For example, for *N* = 5, *n*_*r*_ = 2 and *J*_*rr*_ = 0 we have $E[J_{rr}]=0.4$ and *z*_*rr*_ = −0.82, while *Max*[*J*_*rr*_] = 1. Second, the ratio *n*_*r*_/*N* is high. For example, for *N* = 11, *n*_*r*_ = 10 and *J*_*rr*_ = 8 we have $E[J_{rr}]=8.18$ and *z*_*rr*_ = −0.47. This is obviously highly ordered except that the segments with the same value are separated into two groups. We found also that such cases are due to the presence of one or two empty values (much like the second row in Fig 2).

In rare cases we also observed a number of segments with the same value that have very small number of joins or have no join at all. This is mainly due to the presence of empty values that alternately distributed along a natural street, forming a pattern as shown in the last row in Fig 2. Longer natural streets (usually of higher level) have greater chances of having more empty values. This also explains large differences between *Max*[*J*_*rr*_] and observed joins in S2 Fig. Take Wuhan for example, some high level motorways of the same name are repeatedly segmented by bridges (without a name), leading to a calculated negative *z*_*rr*_ values. To conclude, the negative *z*_*rr*_ values in Fig 5 are not exceptions to but evidence supporting the rule of continuity.

#### Results from Moran’s *I* for speed limits

The ‘maxspeed’ attribute is quantitative data, for which we used Moran’s *I* to quantify its autocorrelation degree along natural streets. In general, the statistics show that for the testing data most (≥ 90%) natural streets appear evidence of positive spatial autocorrelation (except for a few small cities whose ‘maxspeed’ is not populated). For the small proportion which obtains negative Moran’s *I* and *z* values, a detailed look reveals that it is due to the limitation of Moran’s statistics (see below).

**Fig 7:**
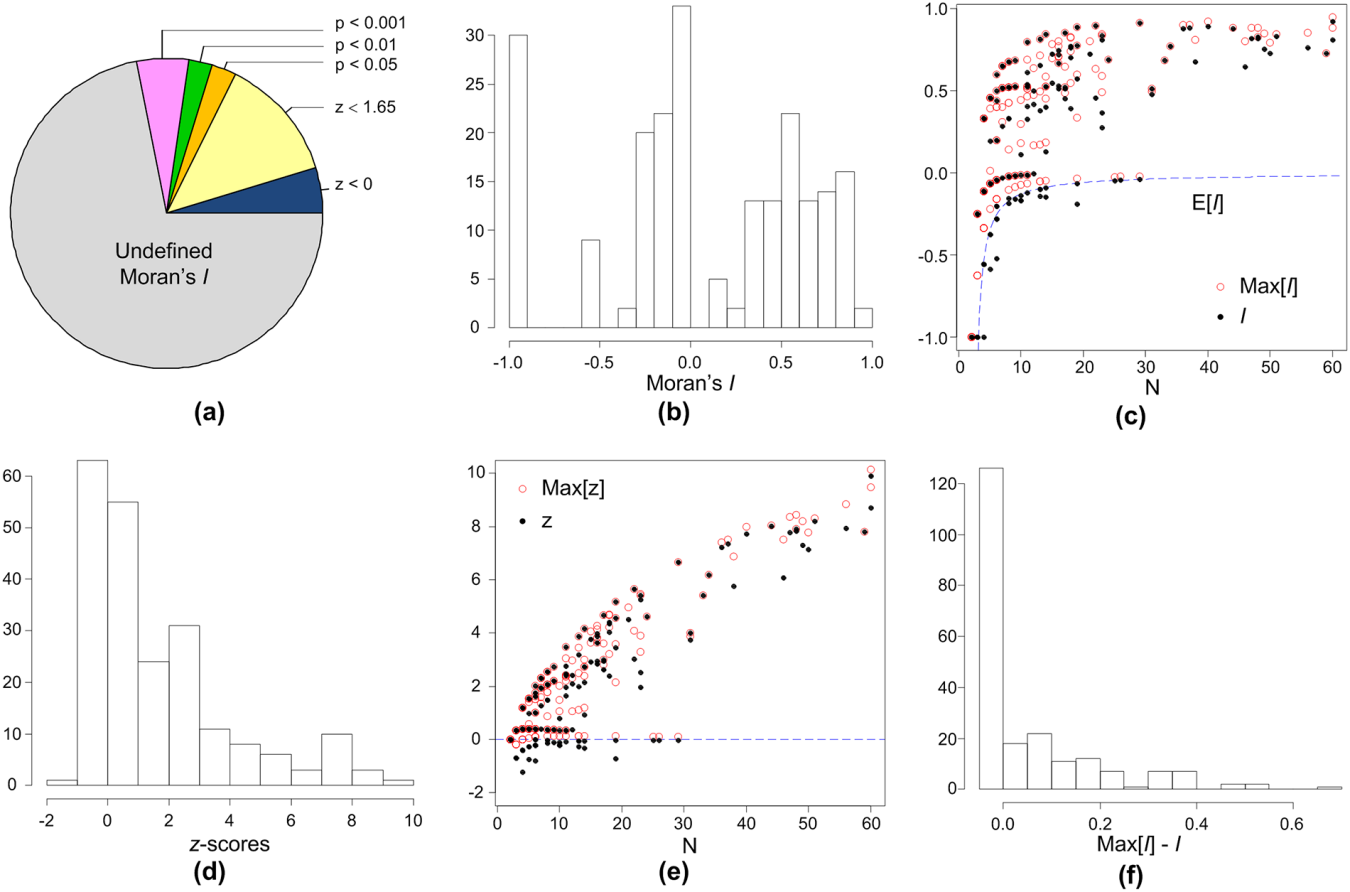
Autocorrelation of speed limits in Geldermalsen of the Netherlands based on Moran’s statistics. (a) resulted Moran’s statistics consist of undefined *I* and *I* values with different *z*-scores; (b) distribution of observed Moran’s *I*; (c) observed Moran’s *I* vs. the maximum *I* for each natural street; (d) distribution of observed *z*-scores; (e) observed *z* vs. the maximum *z* values for each natural street; (f) distribution of distance between observed *I* and maximum *I* (*Max*[*I*] − *I*).

Fig 7 gives a typical view of Moran’s statistics for speed limits along natural streets. For the testing data, a majority of the resulting statistics are undefined *I* values (> 70%), which is because Moran’s *I* is undefined for values with no variance (i.e. all segments in a natural street are of the same speed limits). This is the strongest form of spatial order. Plus the natural streets with *z* > 0, 95% of the natural streets in Geldermalsen shows evidence of spatial order (82.32% of them shows strong evidence with a significance level at *p* < 0.05). Fig 7(b) shows that part of the *I* values are negative. This does not necessarily mean that speed limits on these natural streets are negatively autocorrelated. For short natural streets (e.g. *N* ≤ 5), their maximum *I* values could be below zero depending on what speed limits appear on the natural streets. Note that the maximum *I* values for a natural street is obtained by sorting the values along the street, and we have run permutations of the values which shows that for short natural streets all possible *I* values are below zero. It is apparent in Fig 7(c) and 7(f) that observed *I* values are not very much lower than the max *I* values, and many observed *I* values are still higher than E[I]. This results in the fact that more natural streets obtained positive *z*-scores, implying positive autocorrelation (Fig 7(d)).

By running permutation and sorting on natural streets with negative *z*-scores, we identified two cases. For short natural streets, their maximum *z*-scores are below zero (Fig 7(e)). For longer natural streets we found that, a sequence of equal values mixed with one different value causes the *I* index to give negative *I* and *z* values; as the number of different values increases, the *I* value increases accordingly. This suggests that many natural streets with negative *z*-scores are highly ordered, and that the strength of spatial order for speed limits along the natural street is stronger than what was interpreted from the Moran’s statistics.

#### Universality & exceptions

Our results confirm that strong positive autocorrelation exists for attributes like ‘name’, ‘highway’, ‘ref’, ‘maxspeed’, ‘oneway’, ‘bicycle’, suggesting that the attributes are highly ordered along natural streets. The attribute ‘bridge’ is an exception, where it is negatively autocorrelated for our testing data. That is, bridges seldom stay next to each other but rather are separated along natural streets. For instance, in Beijing we found that *z*_*rr*_ ∈ [−16.12, −0.25] for ‘bridge’ and that no any two bridges stay next to each other, i.e., *J*_*rr*_ = 0 for all segments with a ‘bridge’ tag. In an extreme case, there are 157 bridges along a very long natural street where all of them are separated from each other. This agrees with our intuition that the rule of continuity may not hold for the ‘bridge’ attribute. To summarize, such regularities (both positive and negative spatial autocorrelation) were widely observed in cities of different characteristics.

The testing also shows that segments of empty values are highly autocorrelated. But this can be hard to interpret and is thereby left out from our results. If consecutive segments are really streets without a name, the high autocorrelation confirms the spatial order along natural streets. If on the other hand the empty values are a result of missing values, the high autocorrelation can lead to over-optimistic conclusions, i.e., suggesting a highly ordered structure whereas it is not.

### Rule of symmetry

Our results of parallel road statistics show that a majority (85%±7%) of the parallel pairs are highly similar (with 6-7 attributes having the same value). Table 5 gives a more detailed view on how many pairs of road segments share the same attribute value and for each attribute field. It appears that most attributes, especially those with more controlled vocabularies, give strong support to the rule of symmetry (on average >90% of the parallel pairs are of the same value), except for street names which are populated with free texts. Although we notice that many cities agreed with the symmetry of names, a few cities such as Cario, Paris, Nicosia, and Wellington did not in our initial analysis (‘name’ column in Table 5).

**Table 5:** Evidence supporting the rule of symmetry for OSM data sets. †Proportion obtained by removing parallel pairs with one empty and one non-empty values. ‡Proportion obtained by treating pairs with one empty and one non-empty values as symmetrical examples.

		(%)		proportion of pairs with the same value for the following attributes (%)								
city	# main roads	$\frac{\#\text{empty}\;\text{name}}{\#\text{main}\;\text{roads}}$	# pairs	name	name^†^	name^‡^	highway	ref	maxspeed	oneway	bridge	bicycle
Ahmedabad	2622	73.00	609	84.60	97.00	97.40	88.30	95.40	98.50	55.70	98.20	98.00
Amsterdam	8046	10.10	2602	86.60	91.70	92.20	94.90	92.20	91.80	96.70	97.60	92.50
Athens	10173	17.00	2743	78.60	86.20	87.50	87.20	90.30	83.80	93.10	97.90	97.40
Bangkok	5914	28.90	2605	77.20	89.90	91.40	84.10	86.80	99.80	91.50	93.60	91.80
Barcelona	2460	4.50	718	81.20	83.20	83.60	74.40	82.30	78.40	87.00	98.60	75.20
Beijing	16093	38.90	5568	88.30	96.00	96.30	88.20	94.60	98.40	97.00	96.60	99.70
Cario	4963	56.40	1361	69.10	85.70	88.50	86.50	94.10	96.10	93.10	93.80	99.60
Frankfurt	2920	8.50	1128	89.50	92.70	92.90	95.00	90.40	87.90	99.40	99.00	92.10
Geldermalsen	4356	9.50	412	88.80	95.10	95.40	93.40	92.70	90.00	95.10	97.80	93.40
Hongkong	9287	6.80	3526	78.80	82.40	83.10	85.60	93.00	90.10	95.80	92.60	95.90
Istanbul	3841	80.10	715	82.20	98.70	98.90	83.60	93.70	98.30	84.30	96.80	99.20
London-core	4339	3.80	830	75.70	80.60	81.80	93.00	86.50	93.00	98.00	99.50	89.30
Moscow	17251	16.70	5068	77.10	87.60	89.00	81.60	97.90	83.40	93.90	96.40	97.30
Nagasaki	4851	44.70	325	69.50	83.40	86.20	88.90	86.80	N/A	93.20	95.10	N/A
Nicosia	954	41.00	139	57.60	70.20	75.50	90.60	94.20	98.60	89.20	99.30	100.00
Norwich	3987	1.70	478	80.30	80.30	80.30	86.80	83.30	94.10	94.80	100	97.50
Ottawa	6971	2.90	1615	94.90	95.50	95.50	98.30	97.30	93.60	98.90	99.30	99.00
Paris	4244	5.60	1217	49.20	54.30	58.50	80.40	96.70	85.90	93.20	97.10	92.10
Rio	4040	5.60	1143	70.00	73.20	74.40	73.50	97.90	83.60	95.50	91.50	98.60
San Francisco	48629	3.20	18821	92.20	93.60	93.70	94.90	95.70	92.10	96.50	98.70	95.20
Santiago	7325	3.20	1785	72.80	74.50	75.10	81.90	88.50	91.70	93.70	97.90	99.80
Seattle	16185	6.60	2452	79.60	87.00	88.10	86.40	84.80	81.90	86.50	94.90	88.10
Shanghai	17338	16.60	5851	83.90	89.80	90.50	94.30	94.70	98.30	97.00	96.10	98.00
Singapore	6914	9.90	2840	82.90	89.60	90.40	96.30	95.20	98.00	96.70	97.40	98.20
Sydney	5910	7.60	1118	79.20	80.60	80.90	89.60	94.30	81.90	94.30	97.90	95.30
Toronto	25424	1.20	5584	90.50	91.20	91.30	97.90	100.00	94.00	97.90	98.70	99.30
Wellington	436	16.50	102	66.70	73.90	76.50	81.40	86.30	76.50	90.20	95.10	N/A
Wuhan	6697	47.80	2030	85.30	95.50	96.00	91.60	96.50	99.50	95.60	95.50	99.60

In the following, we discuss the main reasons for this heterogeneity: (1) the proportion of empty values, (2) the performance of our detection algorithm, and (3) the semantic and spelling issues for textual attributes. For each of the reasons we carry out quantitative analysis by eliminating part of the false positives where possible. Finally, insights are drawn from analyzing professional data.

#### Proportion of empty values

The proportion of empty values (with probability *p*) in a data set has a great impact on the result. The attribute values of a pair of parallel roads can be denoted as 〈*v*_1_, *v*_2_〉, where *v*_1_, *v*_2_ ∈ {empty, non- empty}. If we assume a random distribution of empty values among road segments, the probability of observing parallel pairs of different value combinations is outlined in Table 6.

**Table 6:** Probabilities of observing parallel pairs of different value combinations under the random assumption.

value combination of a pair	probability
〈empty, empty〉	*p*^2^
〈empty, non-empty〉	*p*(1 − *p*)
〈non-empty, empty〉	(1 − *p*)*p*
〈non-empty, non-empty〉	(1 − *p*)^2^

For parallel pairs with one empty and one non-empty values which are counted initially as non-symmetrical examples, the probability is 2*p*(1 − *p*) and would go as high as 0.5 when *p* approaches to 0.5. Then, the chances of observing 〈empty, empty〉 and 〈non- empty, non- empty〉 pairs is *p*^2^ + (1 − *p*)^2^, which goes down to its minimum 0.5 as *p* approaches to 0.5, and which goes up as *p* deviates from 0.5. Since parallel pairs with the same (non-empty) value are part of pairs with two non-empty values, the probability of observing the former is even lower. This may help explain why the proportion of parallel pairs with the same name is not high when about half of the name values in the data are empty (e.g. Cario, Nagasaki, and Nicosia). Istanbul is an example of high proportion of empty names (80.1%) that obtains a high proportion of pairs with the same name (82.2%), because it contains many 〈empty, empty〉 pairs.

There are also exceptions like Wuhan and Paris, however. Wuhan has a large number of empty values (47.8%) and still shows good support to the symmetry of names (85.3%). The data consists primarily of rural areas where most divided highways do not have a name (i.e. most of them only have reference numbers such as “S104” which is encoded in the ‘ref’ attribute). This suggests that the random distribution of empty values is a worst case assumption, and that the empty values can be quite ordered reflecting the rule of symmetry. Paris, on the contrary, contains very few empty names (5.6%) but does not show good support to the rule (49.2%) and we will discuss this later.

The reason why our initial result does not show a wide agreement among cities over the world on the symmetry of names (as on the symmetry of other attributes) lies partly in how 〈empty, non- empty〉 pairs are dealt with. In our initial analysis they were taken as non-symmetrical pairs (‘name’ column in Table 5). However, the empty values maybe just missing values due to the quality of user generated content. To get more insights, we did two other calculations: (1) 〈empty, non- empty〉 pairs are removed from consideration due to its uncertainty (‘name^†^’ column in Table 5), and (2) 〈empty, non- empty〉 pairs are counted as symmetrical pairs if we assume that the name of one street in the pair was forgotten by the contributor (‘name^‡^’ column in Table 5). In the two new calculations we see that in general the evidence supporting the symmetry of names become much stronger (87% ± 9% versus 79% ± 10% in the initial analysis). Many cities show significant improvement and are greater than 90%, while for some cities the improvement is limited due to their small proportion of empty names (e.g. Norwich does not show any improvement because it has no 〈empty, non- empty〉 pairs).

Note that ‘ref’, ‘maxspeed’, ‘bridge’, and ‘bicycle’ attributes in OSM street networks have a high ratio of empty values (40%–99%), so the presented strong support to the rule of symmetry may be obtained by chance. This is especially the case for ‘bridge’ and ‘bicycle’ attributes which contain more than 95% empty values. Hence, we cannot draw conclusion as to the strength of the symmetry rule for these attributes simply due to the lack of information in OSM data. On the contrary, ‘oneway’ attribute is populated with less than 8% empty values, and it shows strong support to the rule of symmetry.

#### The algorithm & parameters

A detailed inspection reveals that the recognition contains varying degrees of false positives depending on the data. One important source can be attributed to the fact that there are roads that are not one-way in the parallel pairs. For instance, a two-way road modeled by a single line (i.e. it can be traveled in both directions) can keep parallel to a nearby divided highway, though normally it is not part of it (see S3B, S3D and S3H Fig). We did not remove these two-way roads in our initial analysis for OSM data because we believe that it is not entirely reliable to rely on the tagging system of OSM. To give more insights, we analyzed the data again by removing the single-line two-way roads from the data. This time we focused only on the cities that give weaker support to the symmetry of names, and found that false positives in detected parallel pairs are considerably reduced. The support to the symmetry of names becomes much stronger except for cities like Paris, Rio, and Santiago (Table 7).

**Table 7:** Supporting evidence to the symmetry of street names after removing pairs with a two-way road. †Proportion obtained by removing parallel pairs with one empty and one non-empty values. ‡Proportion obtained by treating pairs with one empty and one non-empty values as symmetrical examples.

		proportion of pairs with the same value (%)		
city	# pairs	name	name^†^	name^‡^
Barcelona	625	83.00	84.50	84.80
Nagasaki	303	79.90	88.20	90.10
Nicosia	124	75.80	89.50	91.10
Norwich	424	89.40	89.40	89.40
Paris	1134	51.50	56.50	60.30
Rio	1085	73.10	75.70	76.60
Santiago	1672	76.10	77.90	78.40
San Francisco	18152	95.10	95.90	96.00
Seattle	2042	90.90	94.40	94.60
Sydney	1054	82.70	84.30	84.50
Wellington	90	72.20	80.20	82.20

Another typical source of false positives happen in complex intersections and highway systems (see S3D, S3F and S3H Fig). Cities like Hong Kong, London and Wellington are typical examples of this. A second source is that, in many situations, more than two road lines lay in parallel to each other, but not every pair of them form a divided highway. For example, roads along the two sides of a river or railway tracks are different roads, but may be detected by our approach as well (e.g. in Rio and Bangkok). These situations reduce the precision of the detection algorithm. If we were able to eliminate the false positives, the supporting evidence would become much stronger for these cities.

Parameters used in our algorithm also determines the performance of the algorithm, which in turn influences the evidence gathered by the algorithm. First, the maximum width of divided highways (i.e. distance between opposite road segments) varies for different cities due to the traffic conditions and also due to the spherical Mercator projection used. As also shown in Fig 4(d), expressways elevated on top of normal roads are modelled by the side of the normal roads, and this makes them unexpectedly wider than they are in reality. As a result, fixed parameter values ($T_{\mu_{dis}}$ and *T*_*cv*_) used in the algorithm may fail to detect elevated divided highways whose width varies from data to data (S3 Fig). The algorithm may also fail when the a double-line highway changes its width along the path (*T*_*cv*_ would increase largely).

#### Semantic and spelling issues in free texts

Another important reason is there exists many semantically similar but literally varied attribute values (e.g. names). For instance, in Paris a divided highway (motorway) has two names *“Boulevard Périphérique Extérieur”* and *“Boulevard Périphérique Intérieur”* on the opposite sides. In total the algorithm detected 240 pairs of such parallel roads which form the outer ring round the city. This explains to a large extent why the symmetry rule is not well observed in Paris (Tables 5 and 7). In Nicosia of Cyprus, there are many instances of such name “Hwy. Nicosia-Troodos” and “Hwy. Troodos—Nicosia” on opposite sides of a motorway.

Additionally, the mixed use of languages and spelling systems is common for OSM data. For Latin languages (e.g. Santiago), Latin and non-Latin characters are used interchangeably in street names. Likewise, street names in Chinese cities can have the following patterns: [Chinese name], [Chinese name + Pinyin], [Pinyin], [Pinyin + English]. Therefore, divided highways in some cities may have different combinations of such patterns on the opposite sides.

These examples actually provide extra support to the rule of symmetry but are counted as non-symmetrical in our initial result. Currently, the semantically identical names were not identified using automated procedures. There are many ways two street names (or other free texts) can read similar to a knowledgeable person, and handling all such cases is computationally non-trivial and therefore out of scope.

Hence we identified some identical names (may not be all of them) manually for a few cities whose support to the symmetry of names is not so strong, and calculated the evidence again: Nagasaki (95.3%), Nicosia (98.1%), Paris (81.5%), Shanghai (94.3%). Besides the super strong support from the rest of the cities, the support from Paris becomes much stronger than it was when identical names are counted as non-symmetrical ones.

#### Insights from professional data

The analysis of OSM data suggests that the rule of symmetry was widely supported for attributes like ‘name’, ‘highway’ and ‘oneway’, where strong evidence was gathered especially after removing some of the false positives in detected parallel streets. No conclusion can be drawn so far as to the other attributes chosen due to the lack of information (i.e. too many empty values).

Hence we tested professional data that are assumed to be consistent and of high quality. Ordnance Survay data has been simplified (compressed) such that dual carriageways are collapsed into single lines and is therefore not suitable for this analysis. Table 8 shows the result for the navigation data set (Nav). The data is filtered such that it contains only one-way road and no empty names. The result confirms a strong support to the rule of symmetry in general. A closer inspection shows that the parallel pairs with different street names are a result of false positives in the detection algorithm.

**Table 8:** Evidence supporting the rule of symmetry in professional data (Nav). ‘class’ is equivalent to ‘highway’ in OSM, ‘structure’ indicates whether a road is bridge or tunnel.

			proportion of pairs with the same value (%)				
city	# road	# pair	name	class	lane	maxspeed	structure
Nav	43009	22829	93.80	93.10	90.40	86.70	99.80

**Fig 8:**
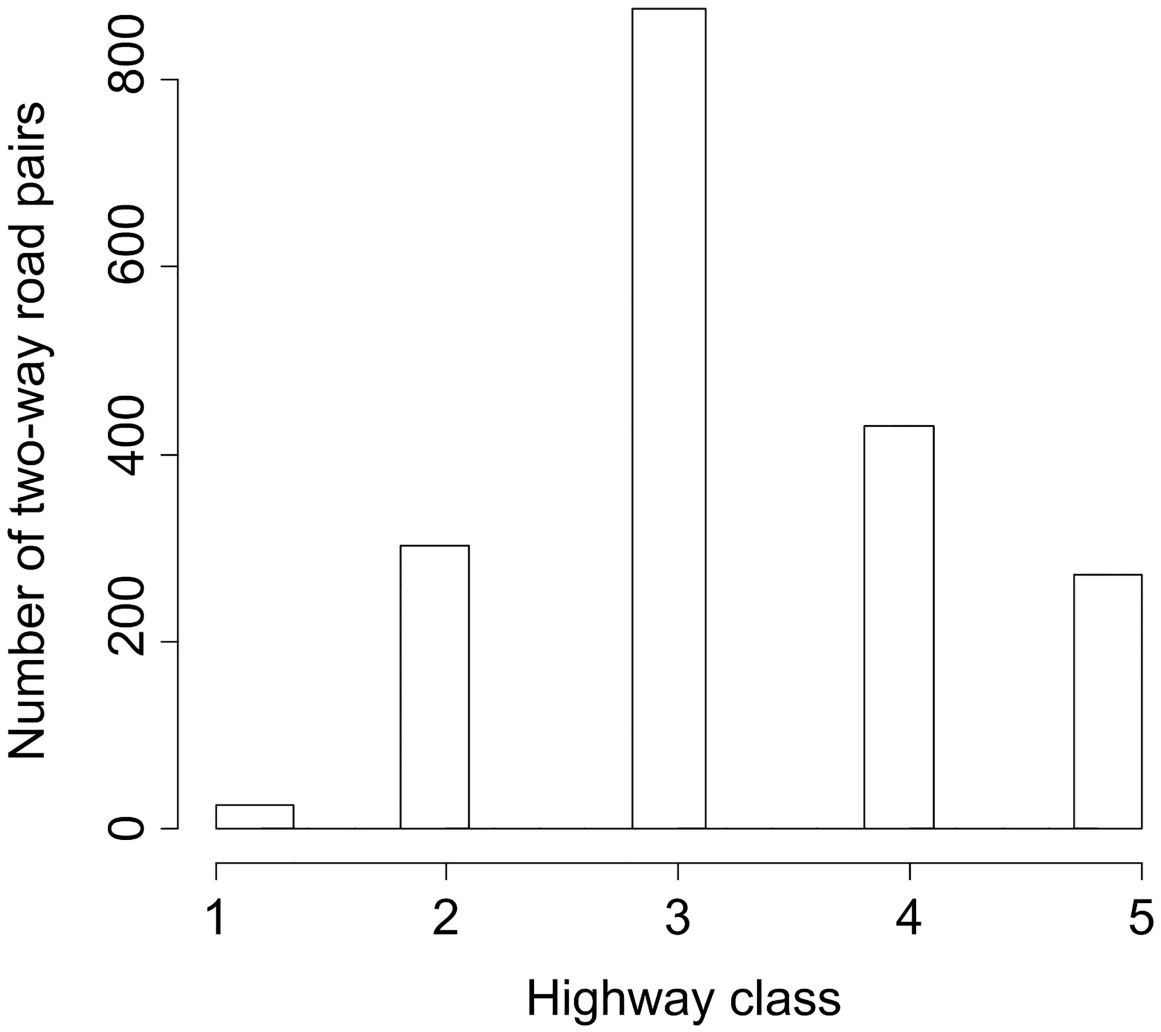
Distribution of highway classes for the two-way roads that have different speed limits on the opposite sides. Classes 1-5 indicate decreasing level of traffic capacity. 1: intercity connections; 3-4: inner city roads; 2: connections between 1 and 3; 5: local networks like residential streets (data: Nav).

In particular, the support to the rule of symmetry for ‘maxspeed’ is not as strong as that for ‘name’, though the evidence (86.7%) is strong enough to be regarded as symmetrical. Since no empty value is allowed for ‘maxspeed’ in this navigation data, the parallel pairs with different maxspeed values must contain exceptions to the rule of symmetry. By excluding false positive pairs (i.e. pairs with different street names), we found that 2428 (out of 22829) pairs of segments have different speed limits on their opposite sides. This is in line with our observations in OSM data, where we identified, though occasionally, cases in which two-way roads have different speed limits on the two sides. We found further that in the Nav data these exceptions mainly occur for mid-class roads (i.e. inner city roads) and less for intercity connections such as motorways and trunks (Fig 8). We suspect that this is because temporary traffic restrictions are common for inner city roads. Similar exceptions occur to ‘lane’, but it is not as prominent as to ‘maxspeed’.

### Possible uses of the rules in quality assurance

These two rules can be used to identify attributes of segments that violate the rules and to further suggest possible corrections. For example, a certain attribute can be segmented into continuous groups by values along a natural street. Within each group a ‘gap’ or ‘spike’ in the value can be a potential missing or inconsistent value, and possible corrections can be suggested according to its surrounding values. This is discussed in more details in Zhang & Ai [32]. The rule of symmetry is more straightforward: if different attribute values appear on the opposite sides of a parallel street, one probably identifies a candidate for inconsistency. In practice, we suggest to framing the use of the two rules in a probabilistic sense, in which possible corrections are notified to human contributors for consideration. The assertion of any inconsistency is better accompanied with a confidence indicator, which varies for different attributes as derived in this paper. This is subject to further research.

### Other forms of spatial order observed

We have also observed a weaker form of the rule of continuity, where road segments connected in local street network (e.g. cul-de-sacs or dead ends) have the same (or semantically identical) name, no matter they are smoothly connected or not. This can be viewed as a relaxed form of spatial order in a network space. In addition, we found that road segments located in the vicinity of other segments of the same name, neither in a natural street nor connected in a local network, indicating a clustering of names that goes beyond the scope of a network-constrained space. However, such patterns cannot be observed constantly in local streets even in the same neighborhood or city, and it is not yet clear under what conditions may such patterns occur. Therefore these observations cannot be used to formulate solid rules in quality assurance yet.

## Conclusion

In order to provide a solid basis for using crowdsourced geographic data (e.g. OSM) in various fields of study or application, researchers and practitioners are particularly concerned with the quality of the data. In this paper we tested two rules that can be used to assess the quality of OSM data. They are the rules of continuity and symmetry and can be thought of as concrete forms of the first law of geography. With these rules, the quality of individual streets in the network can be inspected without referring to ground-truth data. This is important for navigation and location-based services. Automated procedures are proposed to test if the rules are consistent with street network over the world.

Our results suggest that the two rules were widely observed with strong evidence for a selected sample of 28 cities around the world, and for a range of popular attributes. Information (e.g. name, highway class, speed limit, etc.) of street is essentially human-designed and culture-related, but our results observe regularities in continuity and symmetry across cities of different network patterns, sizes, riding conventions, cultures and languagues.

For the rule of continuity, we confirm that most types of information (except for the ‘bridge’) were clustered along smoothly connected natural streets, presenting a high level of spatial order. The every-best-fit strategy is recommended to organize the natural streets. The rule of symmetry was also widely observed, where the ‘maxspeed’ attribute was less well supported than the other attributes; for the symmetry of ‘bridge’ and ‘bicycle’ we cannot draw conclusions due to the lack of information in the OSM data. In practical settings, we suggest using the rules in a probabilistic sense when automatically checking the data consistency and suggesting corrections.

Our methodology can be extended by testing on another set of attributes, or against a different set of data/regions. Note that in our calculation, only textual values that are exactly (literally) the same are considered to be the same. This means that evidence gathered for supporting the rules is still conservative, and can be improved in the future. To use the methodology in detecting the inconsistencies in any practical sense, however, one needs to further improve some of the technical details, e.g., reliably recognizing parallel streets is still highly challenging.

## Supporting information

S1 Fig Distributions of *z*_*rs*_ for typical cities. *z*_*rs*_ for all natural streets are shown in blue; *z*_*rs*_ for selected natural streets (*N* ≥ 10) are superimposed on top of the blue ones (red); red vertical line indicates *z* = 0.
(TIF)

S2 Fig Distributions of *n*_*r*_ − *J*_*rr*_ for typical cities. *n*_*r*_ − *J*_*rr*_ = 1 indicates the strongest form of spatial order.
(TIF)

S3 Fig Typical situations where parallel road detection algorithm gives acceptable and unsatisfactory results. Recognizing divided highways in cases like (g) is highly challenging even for a human subject.
(TIF)

S4 Fig Distribution of *z*_*rr*_ for groups of road segments formed by different strategies. (a) non-continuous random; (b) continuous random; (c) self-best-fit; (d) every-best-fit; (e)-(h) corresponding scatterplot of zrr against N (number of segments in a group/stroke) for each strategy (street data: Nav).
(TIF)

S5 Fig Distributions of the number of segments in natural streets for typical cities. (TIF)
