# Supplementary figures and images for: On the rules of continuity and symmetry for the data quality of street networks
Source: PLoS One. 2018 Jul 12;13(7):e0200334. doi: 10.1371/journal.pone.0200334 (PMC6042734; doi:10.1371/journal.pone.0200334)

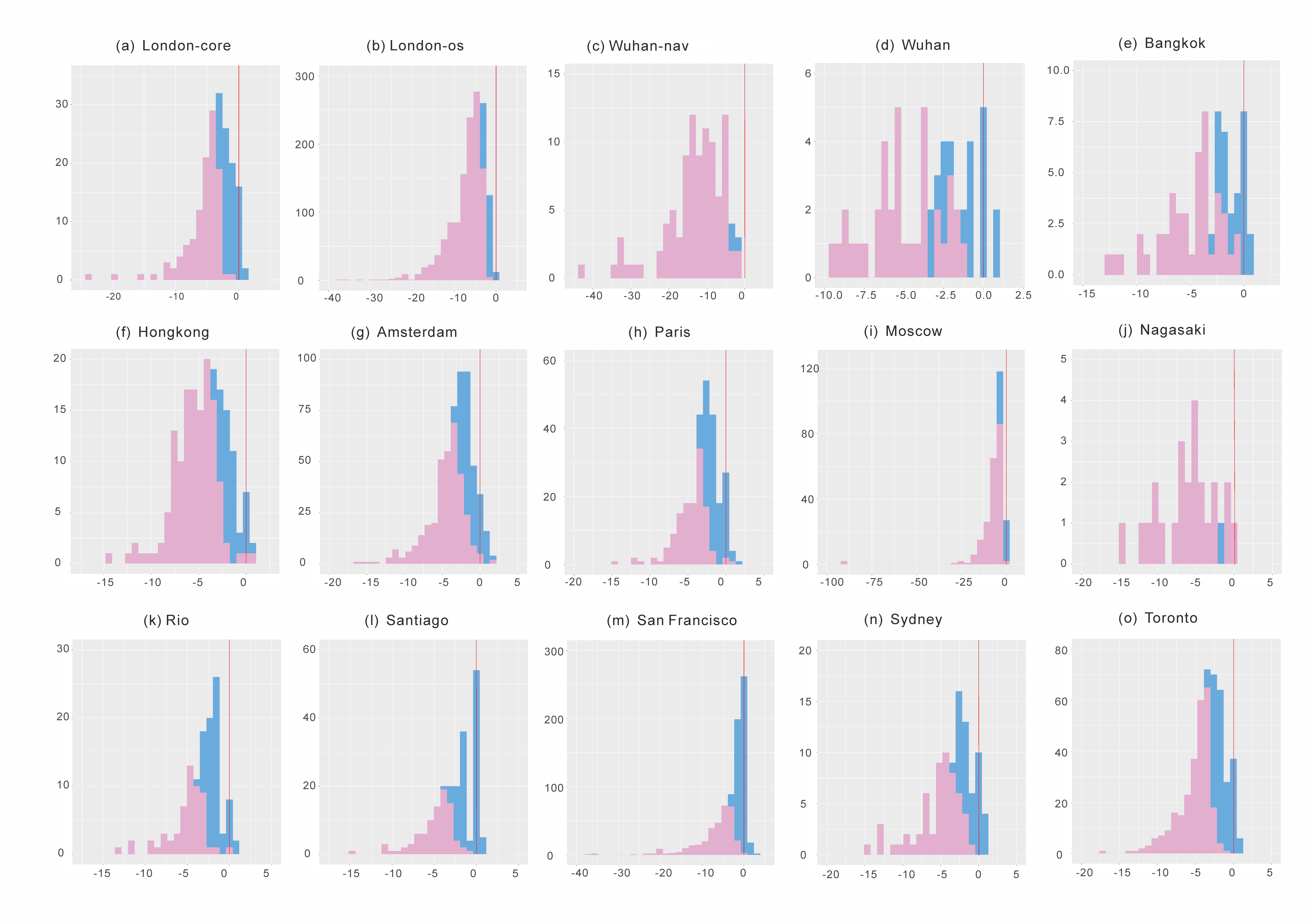

Supplement: S1 Fig — zrs for all natural streets are shown in blue; zrs for selected natural streets (N ≥ 10) are superimposed on top of the blue ones (red); red vertical line indicates z = 0. (TIF) [file pone.0200334.s001.tif]

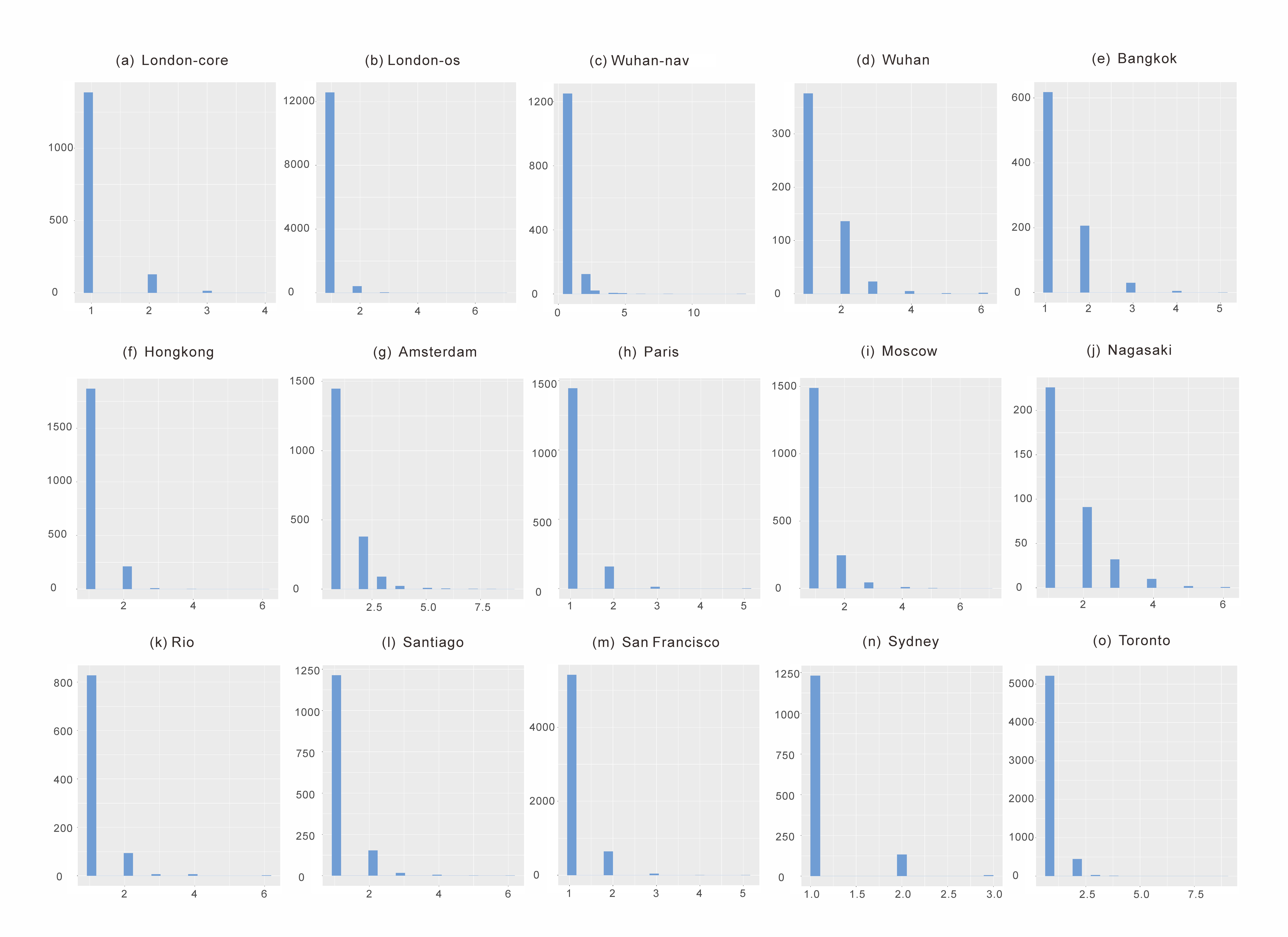

Supplement: S2 Fig — nr − Jrr = 1 indicates the strongest form of spatial order. (TIF) [file pone.0200334.s002.tif]

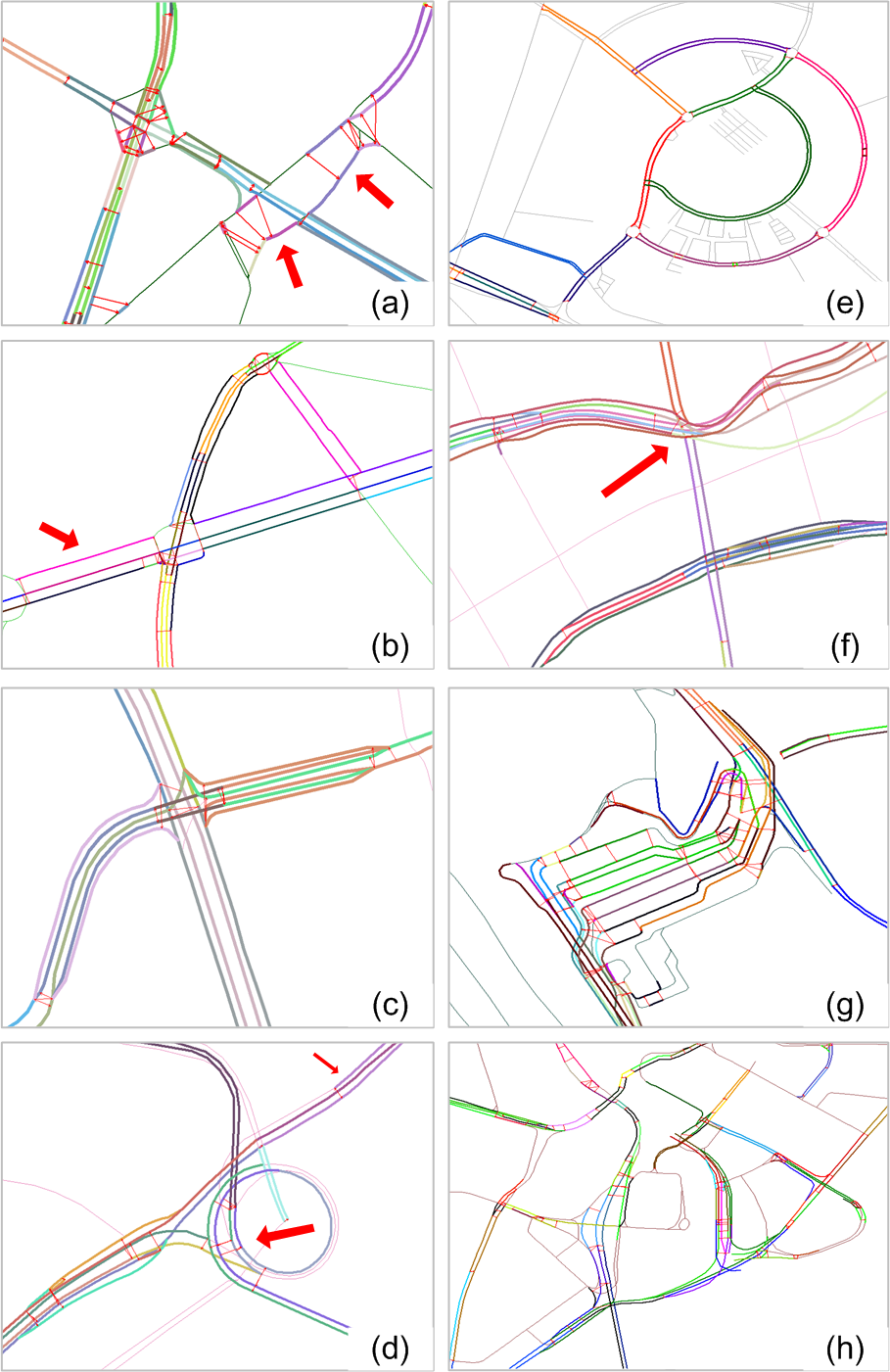

Supplement: S3 Fig — Recognizing divided highways in cases like (g) is highly challenging even for a human subject. (TIF) [file pone.0200334.s003.tif]

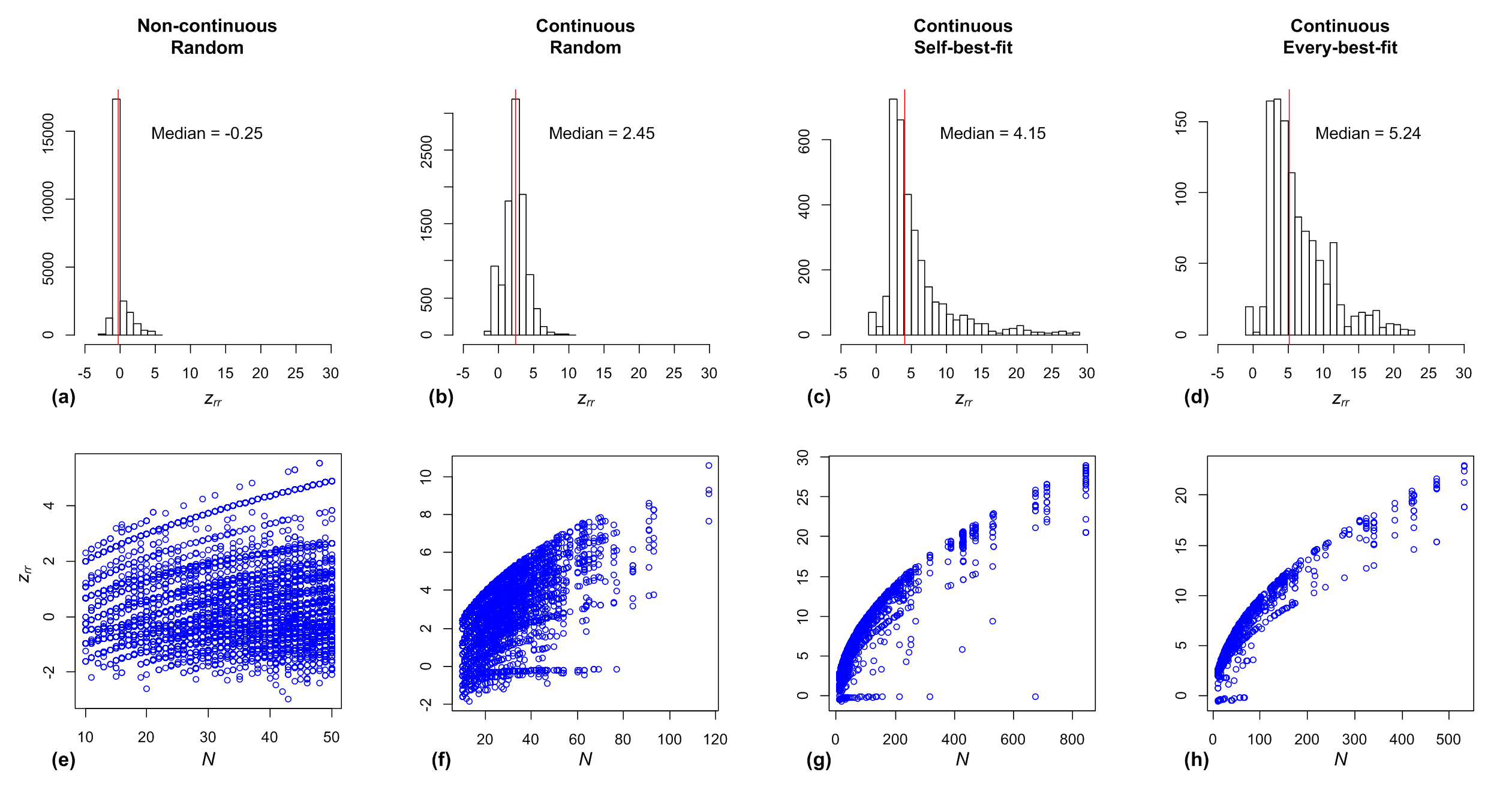

Supplement: S4 Fig — (a) non-continuous random; (b) continuous random; (c) self-best-fit; (d) every-best-fit; (e)-(h) corresponding scatterplot of zrr against N (number of segments in a group/stroke) for each strategy (street data: Nav). (TIF) [file pone.0200334.s004.tif]

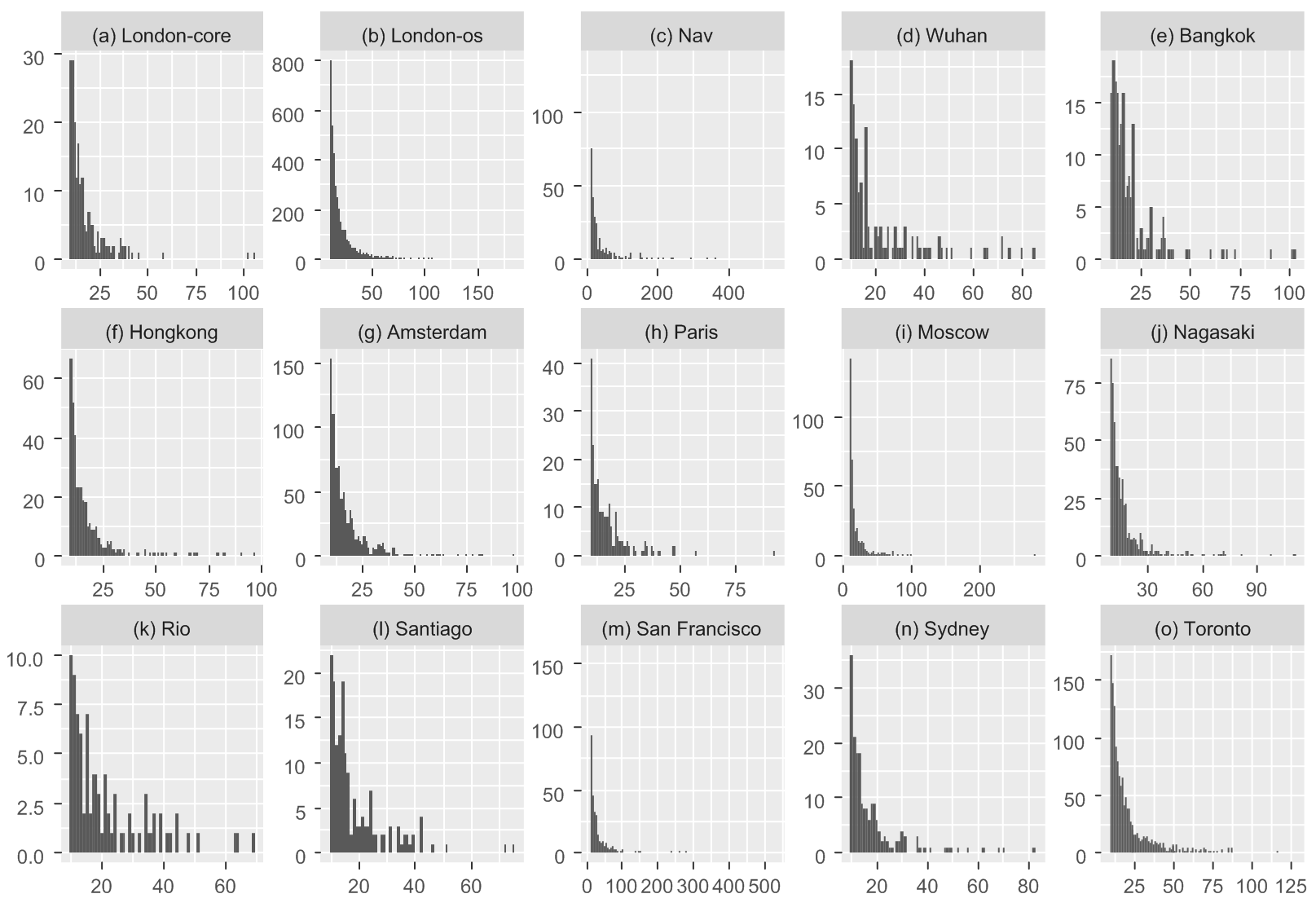

Supplement: S5 Fig — (TIF) [file pone.0200334.s005.tif]
